# Does a Customized 3D Printing Plate Based on Virtual Reduction Facilitate the Restoration of Original Anatomy in Fractures?

**DOI:** 10.3390/jpm12060927

**Published:** 2022-06-02

**Authors:** Seung-Han Shin, Moo-Sub Kim, Do-Kun Yoon, Jae-Jin Lee, Yang-Guk Chung

**Affiliations:** 1 Department of Orthopedic Surgery, Seoul St. Mary’s Hospital, College of Medicine, The Catholic University of Korea, Seoul 06591, Korea; tumorshin@gmail.com; 2 Industrial R&D Center, KAVILAB Co., Ltd., Seoul 06693, Korea; mskim@kavilab.ai (M.-S.K.); dbsehrns@naver.com (D.-K.Y.); hiiamjjinli@gmail.com (J.-J.L.)

**Keywords:** fracture, virtual reduction, 3D printing, patient-specific customized plate

## Abstract

The purpose of this study was to evaluate the restoration of original anatomy after fixation of sawbone fractures using case-specific 3D printing plates based on virtual reduction (VR). Three-dimensional models of 28 tibia sawbones with cortical marking holes were obtained. The sawbones were fractured at various locations of the shaft and 3D models were obtained. The fractured models were reduced virtually and customized non-locking metal plates that fit the reduced model were produced via 3D printing. The fractured sawbones were actually fixed to the customized plate with nonlocking screws and 3D models were generated. With the proximal fragments of the 3D models overlapped, the changes in length, 3D angulation, and rotation of the distal fragment were evaluated. Compared to the intact model (IN), the virtual reduction model (VR) and the actual fixation model (AF) showed no significant differences in length. Compared to the IN, the VR and the AF had mean 3D angulations of 0.39° and 0.64°, respectively. Compared to the IN model, the VR and the AF showed mean rotations of 0.89° and 1.51°, respectively. A customized plate based on VR facilitates the restoration of near-original anatomy in fractures of tibial sawbone shaft.

## 1. Introduction

Fractures are a relatively common injury. In 2019, there were 178 million new fractures worldwide (increased by 33.4% since 1990) and the age-standardized rate of fractures was 2296.2 per 100,000 population [1]. Surgical treatment of fractures has increased along with the increase in the demand for normal functional recovery via anatomical reduction [2,3,4].

Since Hansmann first reported fracture fixation using plate and screws in 1886, plate osteosynthesis has become the standard for fracture surgeries, especially for articular and metaphyseal fractures and some diaphyseal fractures such as those involving the forearm [5]. Fracture plates have evolved over time, and currently anatomically pre-contoured locking plates are widely used [6,7].

However, despite the evolution of the plates, intraoperative anatomical fixation still entails manual reduction of the fracture, followed by plate contouring because a single anatomical plate does not fit all the patients. Patient-specific fracture plates may overcome such inconvenience but they are still to be developed, although various patient-specific treatments employing three-dimensional (3D) printing technology have recently been used. The lack of a model for case-specific plate design is one of the primary hurdles in this area.

In this study, we used the virtual reduction (VR) of a fracture as the basis for designing customized plates. The purpose of this study was to evaluate the restoration of original anatomy after the actual fixation of sawbone fractures using 3D printed plates based on VR.

## 2. Materials and Methods

### 2.1. Experimental Procedures

**Figure 1:**
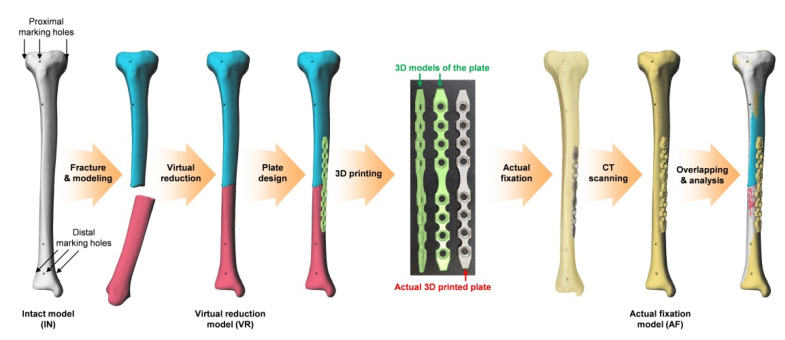
Overall experimental flow. Three-dimensional, (3D).

A total of 28 tibial sawbones (ORTHObones model W19122, W19126, W19129, and W19131, size 15.4 in × 31.9 in × 2.8 in, weight 0.35 kg, 3B Scientific, Hamburg, Germany) were used, and the overall experimental flow is depicted in Figure 1. In each sawbone, cortical marking holes were drilled to consistently localize the same measurement points on the 3D models obtained by multiple scanning. Three cortical marking holes were drilled in the same proximal axial plane and another three holes were drilled in the same distal axial plane using a customized jig. Each one of the three holes was located in the mid-sagittal plane of the tibia. Following computed tomography (CT), 3D models of the sawbones were obtained using MIMICS software (Ver 21.0, Materialise, Leuven, Belgium); these models were categorized as intact (IN). Using this software, the 3D coordinates of the center of each marking hole were automatically calculated so that subsequent measurements could be performed without observer bias.

**Figure 2:**
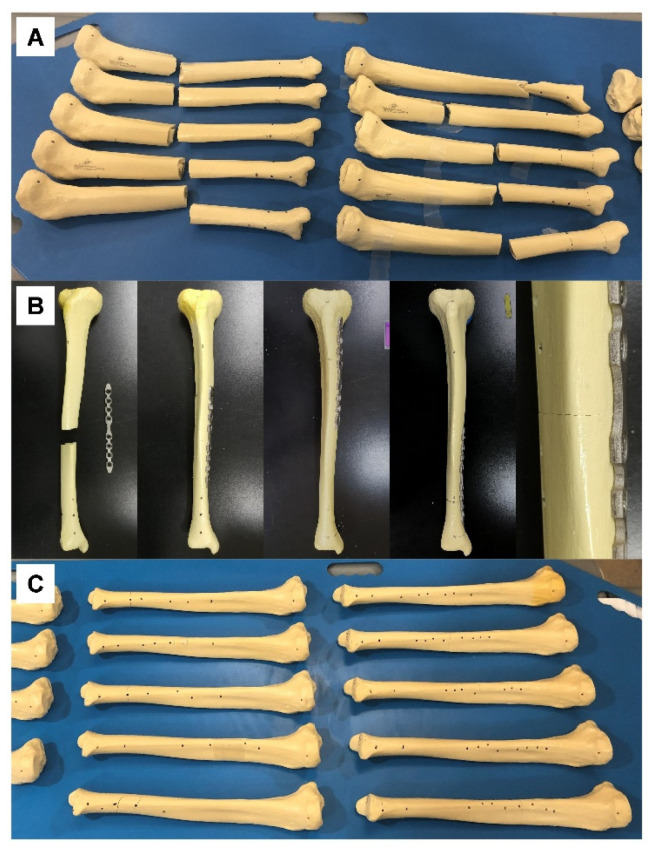
(**A**) Tibial sawbones fractured at various longitudinal locations of the shaft, prior to CT scan. (**B**) Sawbones and customized plates prior to fixation (left image in row), fixed at various locations (middle three images in row), and enlarged at the fracture site (right image in row). (**C**) Tibial sawbones actually fixed with a customized plate based on virtual reduction, prior to CT scan.

The sawbones were fractured at various longitudinal locations of the shaft (Figure 2A), and 3D models were obtained after CT scan. The fractured models were reduced virtually using Metasequoia 4 software (Ver 4.7.0, Tetraface, Tokyo, Japan); these models were grouped under the virtual reduction (VR) category.

Using the same software, a case-specific fracture fixation plate with four screw holes for proximal fragment and four screw holes for distal fragment was designed to fit the medial surface of each VR model. The contact surface (with bone) of the plate was designed according to the Boolean function in Metasequoia 4 to ensure perfect contact between the bone surface and the plate. The plates were then fabricated using a powder bed fusion type 3D printer (DMP 350, 3D Systems, SC) and titanium powder (grade 23 Ti-6AI-4V alloy). Post-processing consisted of removal of the supporter, surface finishing using a hand piece, and blasting with ceramic microbead. The dimensions of the plates were 149.5 ± 1 mm in length, 12 ± 0.1 mm in width, and 2.5 ± 0.1 mm in thickness. The actual fractured sawbones were reduced and fixed to the plate with nonlocking screws to ensure complete contact of the fragments with the customized plate (Figure 2B). Three-dimensional models of the fixed sawbones obtained after CT scan were grouped under the actual fixation (AF) category.

### 2.2. Evaluation of the Alignment

**Figure 3:**
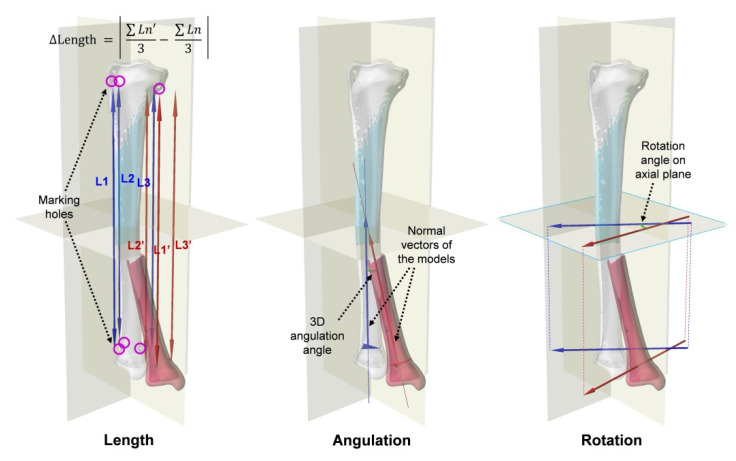
Evaluation of the alignment with the proximal fragment of the models overlapped: length (the mean distance between marking holes in longitudinal axis), angulation (3D angle between normal vectors of the distal fragments), and rotation (the angle between the axial plane projections of the vectors passing through two of the three distal marking holes).

To evaluate the alignment after VR and AF, the IN, VR, and AF models of each sawbone were overlapped virtually in 3D space while ensuring the same position of the proximal fragments of each model (Figure 3). Sagittal, coronal, and axial planes were set to fit the shape of the tibia. The alignment was evaluated based on three parameters: length of the model, angulation of the distal fragment, and rotation of the distal fragment.

The length of each model was defined as the average distance in the longitudinal direction between the centers of the marking holes proximal 3 and distal 3. The lengths of the IN, VR, and AF models were compared. The differences in the length of VR compared to that of IN and the length of AF compared to that of IN were also measured. The angulation of the distal fragment was defined as the 3D angle between the normal vector of the distal fragment of IN and that of VR or AF. The normal vector of the distal fragment was obtained from the plane containing the centers of three distal marking holes. The degree of angulation for VR and AF was compared. The rotation of the distal fragment was defined as the angle between the axial plane projection of the vector passing through two of the centers of the three distal marking holes of IN and that of VR or AF. The degrees of rotation for VR and AF were also calculated and compared.

### 2.3. Statistical Analysis

Statistical analyses were conducted using Statistics and Machine Learning Toolbox on MATLAB (R2021b, MathWorks, Natick, MA, USA). Descriptive statistics are presented as mean (M) and standard variation (SD). The significance of the differences in the mean values of each evaluated parameter was analyzed via paired *t*-tests after verifying the normality of the data distribution. The significance level was set at *p* < 0.05.

## 3. Results

**Figure 4:**
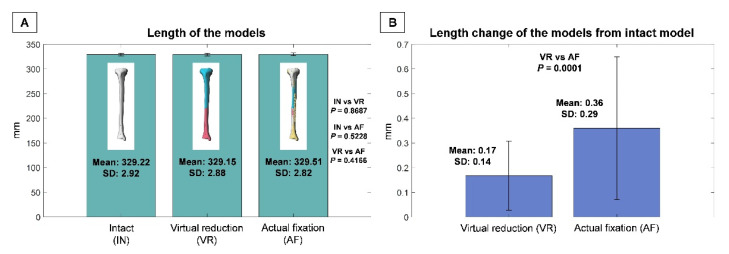
(**A**) Lengths of intact (IN), virtual reduction (VR), and actual fixation (AF) models. (**B**) Length variation of VR compared to IN and that of AF compared to IN. Standard deviation, (SD).

The mean lengths of the IN, VR, and AF models were 329.22 mm (SD, 2.92 mm; range, 323.50–336.21 mm), 329.15 mm (SD, 2.88 mm; range, 323.66–336.03 mm), and 329.51 mm (SD, 2.82 mm; range, 324.05–335.92 mm), respectively (Figure 4A). There were no significant differences in length between any two of the three models (*p* > 0.05). The mean length difference (either lengthening or shortening) of VR relative to IN was 0.17 mm (SD, 0.14 mm; range, 0–0.81 mm), which was significantly greater than that of AF relative to IN (0.36 mm, SD, 0.29 mm; range, 0–1.32 mm) (*p* = 0.0001) (Figure 4B).

**Figure 5:**
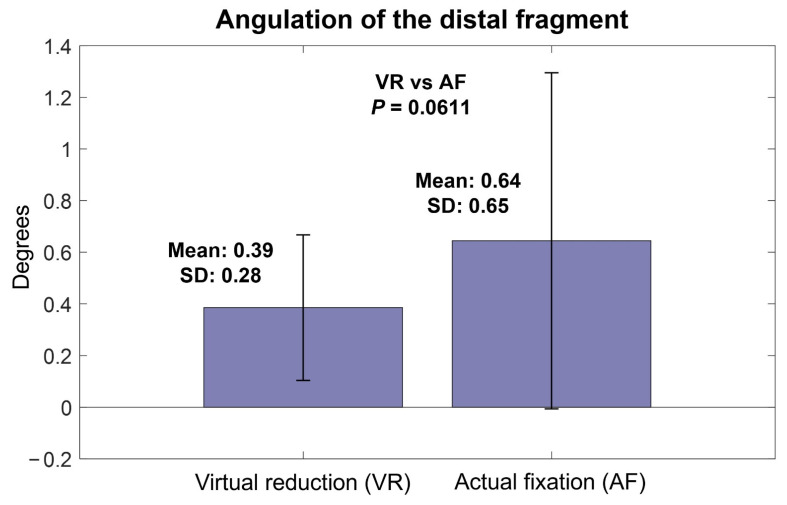
Angulation of virtual reduction and actual fixation models relative to intact models.

**Figure 6:**
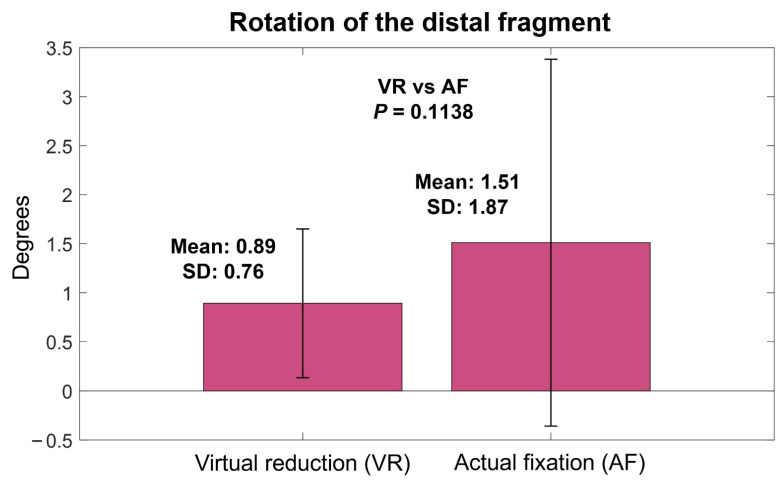
Rotation of virtual reduction and actual fixation models relative to intact models.

The mean angulation of VR relative to IN was 0.39° (SD, 0.28°; range, 0–0.92°), which was not significantly different from the mean angulation of AF relative to IN (0.64°, SD, 0.65°; range, 0–3.01°) (*p* = 0.0611) (Figure 5). The mean rotation of VR relative to IN was 0.89° (SD, 0.76°; range, 0.09–3.34°), which was not significantly different from that of AF relative to IN (1.51°, SD, 1.87°; range, 0–9.54°) (*p* = 0.1138) (Figure 6).

## 4. Discussion

The principal finding of the current study is that a fractured bone can be reduced to near its original anatomy with a customized plate based on virtual reduction. The tibia was chosen for this study because it has sufficient length and width on which to place the marking holes far from each other so that the alignment could be measured accurately. The fracture was made at various longitudinal locations of the tibial shaft to simulate various innate anatomy features (curvatures and twists on the surface) at the fracture site. The acceptable range of fracture reduction alignment is generally evaluated by length, angulation, and rotation [8,9]. For a tibia fracture, 10–20 mm shortening, 5–10° angulation, and 10–20° rotation are considered to be acceptable [10]. Tibial malunion occurs in 3–50% of conservative treatment and up to 20% of surgical fixation [10]. Although plate osteosynthesis has the lowest malunion rate for tibial fractures [11,12,13], the malunion rate reaches up to 8.3% even after tibial plating [14], and midshaft fractures do not have a lower malunion rate than distal fractures [15]. The mean angulation after tibial plating reaches up to 2.6–3.1° even on a single coronal plane [16,17]. In our results, both VR and AF showed minor mean changes in length (0.17 and 0.36 mm), angulation (0.39 and 0.64°), and rotation (0.89 and 1.51°) compared to the pre-fracture condition. These results suggest that the alignment effect of the VR-based plate itself is excellent, although further studies are needed to investigate whether the final alignment will equal the alignment immediately after fixation in clinical practice.

At present, fracture surgery using a metal plate involves manual reduction of the fragments followed by manual contouring of the plate to fit the reduced bone. Sometimes the manual reduction itself is difficult and may require alignment verification with intraoperative radiographs because the operator can only see the exposed part of the bone. Even with successful reduction, repeated manual bending and twisting of the plate is necessary to fit the plate to the reduced bone. Although contemporary locking plates do not need to fit to the bone for stability [18,19], at least some manual contouring is still necessary, even for anatomical plates, to install the plate and screws in the right position and prevent plate protrusion or irritation. These procedures require additional surgical time, effort, and equipment. The outcomes of the fixation may vary depending on the operator’s experience or know-how. Further, there are cases in which a plate does not fit the bone despite bending and twisting, or an important fragment cannot be purchased because the screw holes are limited to certain fixed locations.

To address these limitations, studies have utilized 3D printing technology in fracture surgeries. The application of 3D printing in fractures varies widely from 3D modeling for screw fixation to customized casting [20,21]. The existing works on 3D printing fracture plates can be largely divided into tactile modeling and mirroring of the normal contralateral bone. Recent reports of tactile modeling techniques have involved real-size 3D printing of the fractured bone for pre-practicing of the surgery and pre-contouring of the plate [22,23,24]. Although tactile modeling reduces intraoperative time, blood loss, and fluoroscopies [25], the overall procedure still entails manual reduction of the tactile model and bending and twisting of the plate by the operator.

Meanwhile, the other type of approach utilizes mirroring of the normal contralateral bone. Various authors have used mirrored models to pre-contour a ready-made plate and achieved good outcomes [26,27,28]. A recent study reported 3D printed case-specific pelvic fracture plates based on a mirrored 3D model of the contralateral pelvis [29]. When using such a strategy, manual pre-reduction of the fracture is not necessary to pre-contour the plate. However, in practice, there are cases in which a normal contralateral image is not available, such as cases involving bilateral fractures or when the patent refuses to undergo an additional CT of the uninjured other side. The difference between right and left bones may also be problematic and may lead to malalignment after reduction or protrusion of the plate. A poorly-fitting 3D printed plate may be especially problematic because it is still difficult to ensure both strength and ductility of 3D printed metals [30,31] and bending of the 3D printed plate may result in fracture.

However, in this study, we used a virtually reduced model as the basis of each case-specific fracture plate and thus achieved near-original alignment. Our results suggest that the virtually reduced bone represents an excellent model for designing case-specific fracture plates. The VR has advantages over intraoperative manual reduction in which the operator only visualizes the exposed area of the bone or has to perform fluoroscopy. Further, compared to the mirror method, the VR does not result in errors caused by differences between right and left bones, and it does not increase the cost or radiation exposure during contralateral imaging.

In addition to the excellent fit, the VR-based customized plate has an advantage in which the plate itself can guide fracture reduction intraoperatively. Because the plate is designed to fit the reduced model, the operator can both reduce the fracture and determine the alignment by attaching the bone fragments to the plate. In conventional fracture fixation, the operator manually reduces the bone, determines the alignment, and temporarily clamps the fragments to hold the reduction. Then a plate is placed on the surface of the bone, and is bent and twisted accordingly. These procedures may require considerable amounts of intraoperative time and effort. In addition, sometimes the clamp holding the reduction hinders the attachment of the plate onto the bone to determine the fit. By contrast, our method involved screwing the fragments to the plate with nonlocking screws to ensure complete contact. The procedure was simpler and restored almost original anatomy of the fractured sawbones. This may allow less-experienced surgeons to operate easily and quickly, reduce radiation exposure and need for related equipment, and prevent plate-related complications such as plate protrusion or irritation. In addition, good contact between the bone and the plate may strengthen the fixation and facilitate better alignment in minimally invasive plate osteosynthesis.

However, the VR-based plate is not completely free from performance-related errors, in that the mean length of VR model was shorter than that of IN model, and in that the length difference of AF was significantly greater than that of VR. In some cases, the task performer for the VR had to let the virtual fragment slightly intrude into the other virtual fragment to obtain better overall alignment. This made the mean length of the VR model shorter, although this difference was not statistically significant. The significantly greater length difference of AF can be attributed to the surgical technique. The angulation and rotation were also greater in AF than in VR, although this difference was also not statistically significant. For example, compression across the fracture site due to eccentric placement of the screws resulted in a slight gap between the far cortices at the fracture site (Figure 2). Although the overall final alignment was excellent in our study, there is room for achieving even better alignment with the additional use of appropriate techniques such as centering the screws in the holes while using the customized plate. Designing the plate slightly concave to the bone to allow compression across the fracture site may also improve the final alignment. Further studies are needed to investigate these possibilities.

The current study has limitations. First, no comparisons were performed with conventional or mirroring-based plates. However, the final alignment parameters were adequate to ensure fracture surgeries. Second, a single type of sawbone was used, and relatively simple fractures were simulated. Although we simulated the individuality of each bone shape and fracture type by making the fractures at different longitudinal locations, additional studies involving various bones and complex fractures are needed to further confirm the reliability of VR. Third, this study was an experimental study, and it did not take into account in vivo conditions such as the soft tissue or the bone quality. In practice, the final alignment of the bone can be affected by soft tissue tension or low bone density. In vivo evaluations based on the present work are necessary to further investigate the clinical relevance of the customized fracture plates. However, in practice, the original anatomy is missing when fracture patients visit a clinic, which means there is a need for an experimental study to accurately evaluate the aligning effect of the customized plate. For such a study, we believe that a sawbone experiment is the best because, in an in vivo experiment, the soft tissue or the bone quality may confound the aligning effect of the customized plate itself. Lastly, before our method can be actually applied in fracture surgeries, further studies are needed to reduce the time needed for image engineering and manufacturing, and to ensure the mechanical properties of the customized plates.

In conclusion, a customized plate based on VR facilitates the restoration of near-original anatomy in fractures of tibial sawbone shaft. Besides the excellent fit, the plate itself can guide the alignment of fracture reduction. The use of a VR model as the basis of implant design may facilitate the application of 3D printing technology in personalized fracture surgeries.

## Data Availability

The data presented in this study are available on request from the corresponding author.
